# Supplementary figures and images for: A mathematical representation of protein binding sites using structural dispersion of atoms from principal axes for classification of binding ligands
Source: PLoS One. 2021 Apr 8;16(4):e0244905. doi: 10.1371/journal.pone.0244905 (PMC8031081; doi:10.1371/journal.pone.0244905)

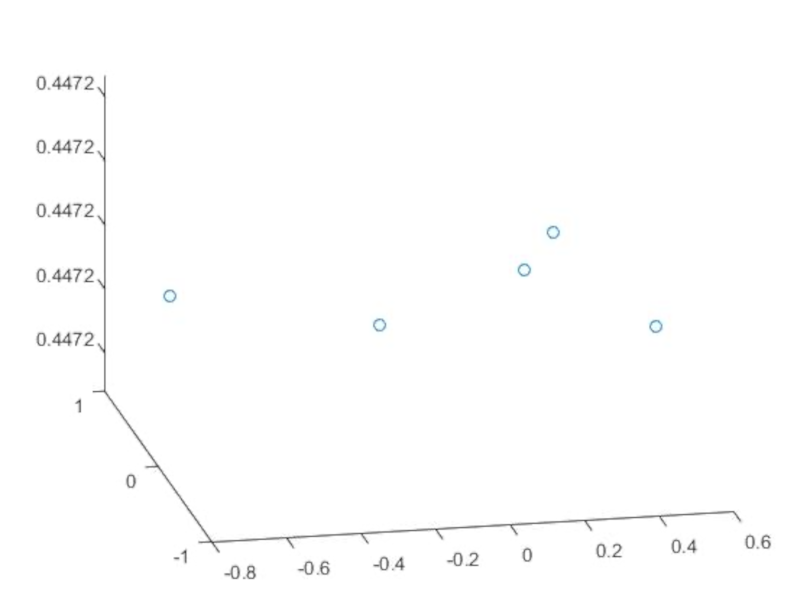

Supplement: S1 Fig — If even a single observation is removed, the mean and covariance structure for the entire group will be radically altered, which makes leave-one-out cross validation fail. (TIF) [file pone.0244905.s001.tif]

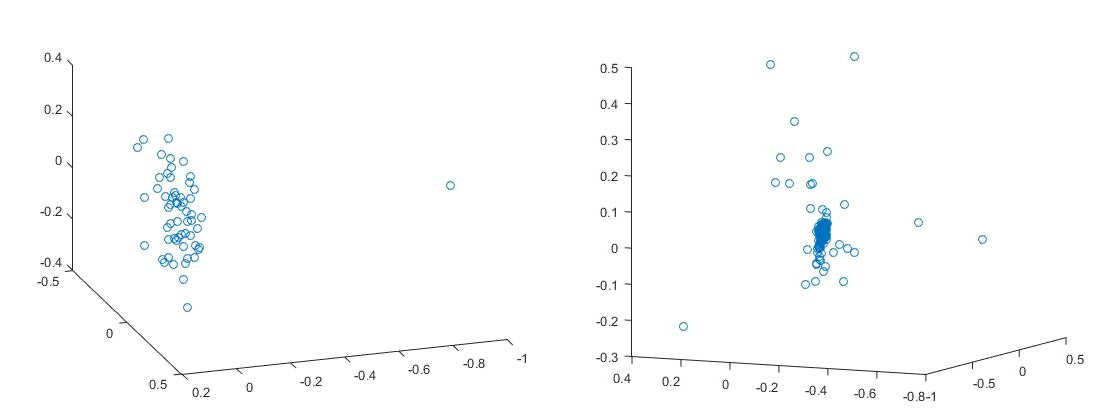

Supplement: S2 Fig — (Left) For the FMN group (Right) For the PO4 group. (TIF) [file pone.0244905.s002.tif]

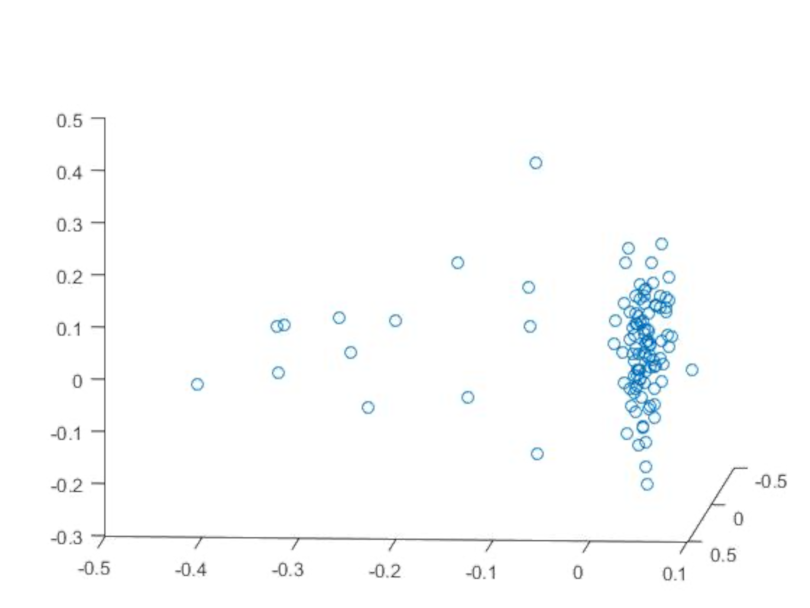

Supplement: S3 Fig — (TIF) [file pone.0244905.s003.tif]

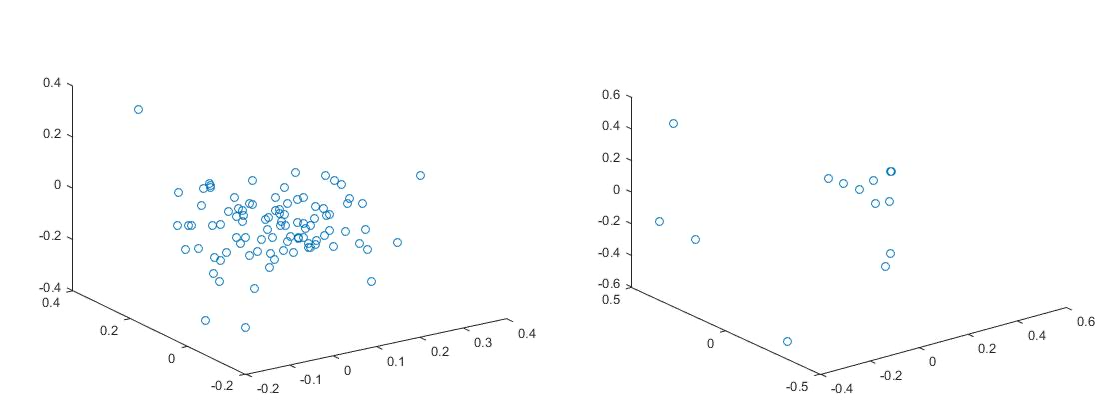

Supplement: S4 Fig — (Left) For ligand HEM-I. (Right) For ligand HEM-II. (TIF) [file pone.0244905.s004.tif]

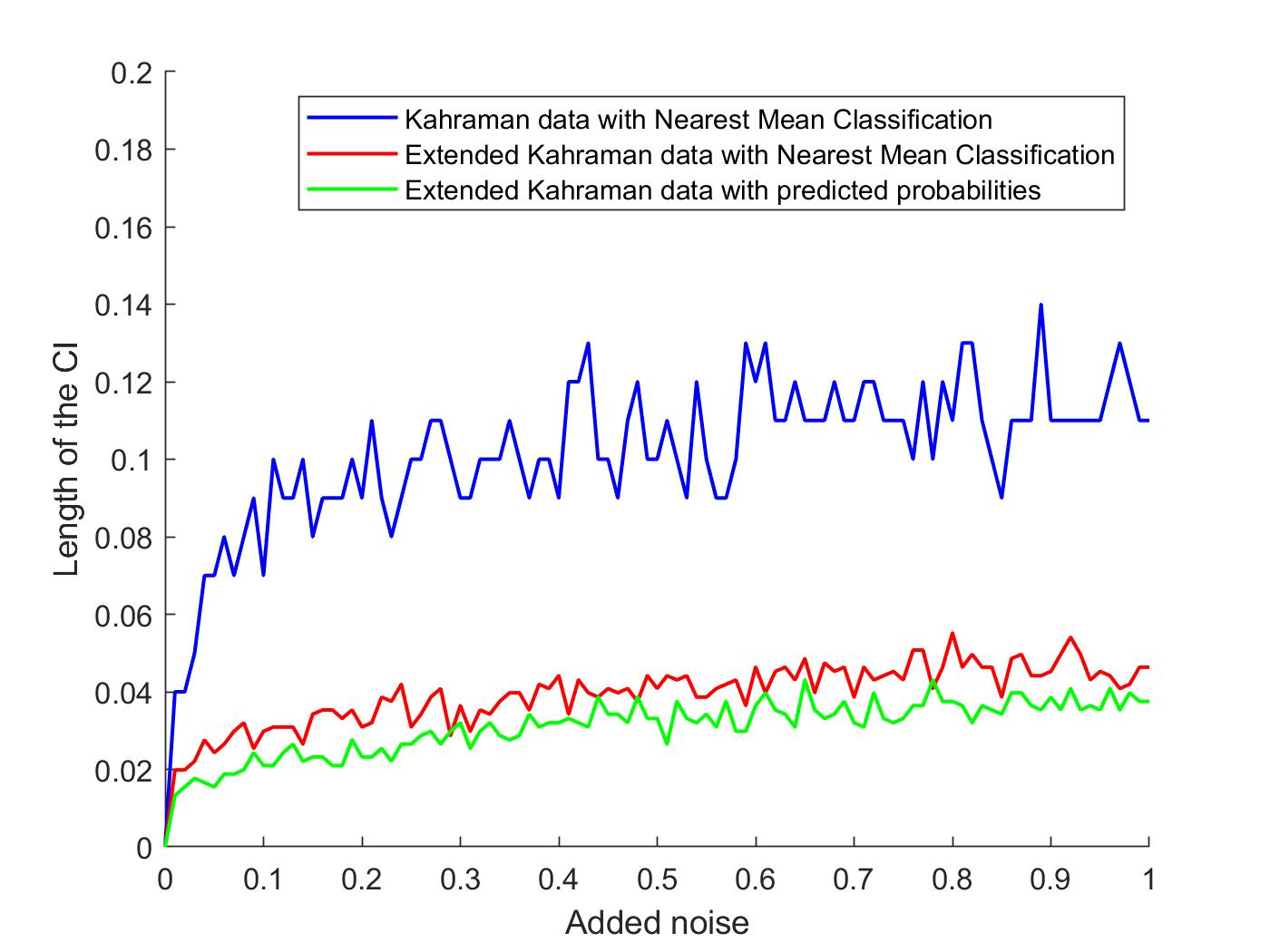

Supplement: S5 Fig — (TIF) [file pone.0244905.s005.tif]
